# Manual of Midwifery

**Published:** 1871-12

**Authors:** 


*Manual of Midwifery.* By Alfred Meadows, M. D., Lond. Philadelphia: Lindsay & Blakiston, 1871.

In looking hastily over the contents of this work we find that it is a pretty complete work upon obstetrics; few subjects in the whole field, but have passed under the notice of the author. It is concise, well arranged and remarkably complete, and as a guide to the student in his lecture term, and as a ready reference to the physician, no work of similar character equals it in value.

